# Contrasting Patterns of Hemarthroses in Different Joints After Development of Hemophilic Arthropathy in a Severe Type A Hemophiliac: An Autobiographical Case Report

**DOI:** 10.7759/cureus.20947

**Published:** 2022-01-05

**Authors:** Abhishek Haryani, Akansha Sastya, Daulat Ram Haryani

**Affiliations:** 1 Medicine, Mahatma Gandhi Memorial Medical College, Indore, IND; 2 Internal Medicine, Mahatma Gandhi Memorial Medical College, Indore, IND; 3 Pediatrics, Private Clinic, Indore, IND

**Keywords:** hemarthroses, autobiographical case report, neoangiogenesis, arthritis, synovitis, hemophilia, arthropathy

## Abstract

Hemophilic arthropathy is a very common complication in patients suffering from severe hemophilia A (factor VIII activity level <1%). It is the most common cause of long-term joint damage and reduced mobility in people with hemophilia A. This case report describes the author's experience with different and contrasting changes in hemarthroses (bleeding episodes in joints) after developing arthropathies in said joints. While both the knees and the right elbow have severe arthropathy and arthritic changes, the hemarthroses in the knees have decreased while they have increased in the right elbow. This contrast can be attributed to changes in musculature and vasculature around the joint.

## Introduction

Hemophilic arthropathy is a degenerative/depositional joint disease occurring in hemophilic patients as a long-term consequence of repeated hemarthroses in the joint space. Around 50% of patients with hemophilia will develop severe arthropathy. Clinically, it presents similar to osteoarthritis, with a reduced range of function and movement, chronic joint pain, stiffness, and frozen limbs (especially in the morning), gradual muscle weakening, and loss of vasculature around the joint. Some joints will show decreased bleeding episodes due to reduced vasculature around the joint in advanced arthropathy [1].

This case report describes the author's experience with contrasting bleeding patterns in the knees and right elbow. As the literature surrounding this is scarce and non-existent, the author wanted to present an opportunity for other medical professionals to better understand the changes surrounding bleeding habits in a hemophilic patient with joint disease.

## Case presentation

Early life

I was diagnosed with severe hemophilia A in 1999, at the age of 1. My factor VIII level was less than 1% of the normal range. I have suffered from multiple episodes of bleeding every year since the diagnosis. The target joint (the joint with the most bleeds) was my right knee for most of my life, and I developed a slight flexion deformity in the right knee. Other joints frequently affected were the left knee, right elbow, and left ankle. The bleeding episodes have been treated with recombinant factor VIII infusions since I was two years old, and I have never received a plasma transfusion as a form of treatment. Currently, I am on a prophylactic treatment, receiving six recombinant factor VIII infusions every month.

Management of frequent hemarthroses

Due to multiple joint bleeds monthly and a continued decreased quality of life, I had a yttrium radiosynovectomy in 2011 and 2012 in my right and left knee, respectively. It was an outpatient procedure done under local anesthetic. After both procedures, I was bed-ridden for three days, following which I started progressive physical therapy, which would help me regain muscle strength and mobility. Both the radiosynovectomies led to fewer bleeding episodes. Before the procedure, I would get 8-10 bleeding episodes every year in both knees, and after the procedure, I had only two to three bleeding episodes a year in both knees. There was a drastic improvement in the quality of life. It helped me get regular and better physical therapy for joints affected by hemophilia without being interrupted by constant hemarthroses episodes.

Development of arthropathy

In late 2018, I started observing decreased mobility and increased stiffness in both my knees. My walking pattern changed and worsened. It became extremely difficult to walk right after I stood up from a chair or bed. These symptoms become progressively worse with time.

**Figure 1:**
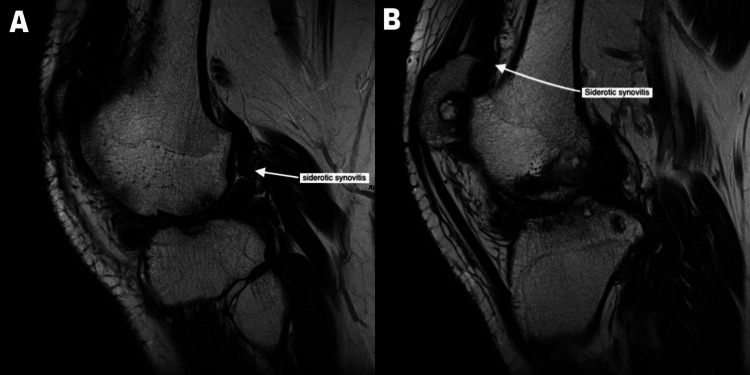
Siderotic synovitis MRI Images showing siderotic synovitis

**Figure 2:**
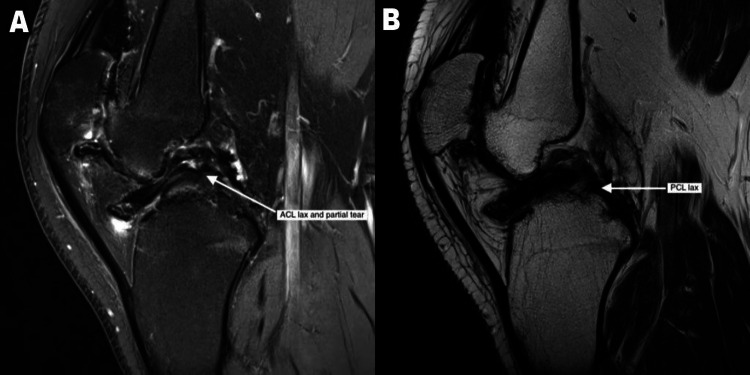
ACL showing lax and partial tear and PCL showing lax (A) ACL, MRI image showing lax and partial tear; (B) PCL, MRI image showing lax. ACL: anterior cruciate ligament; PCL: posterior cruciate ligament.

In June 2019, I had an MRI on suspicion of developing the joint disease. The MRI had many findings suggestive of joint degradation. The knee joint showed mild flexion deformity and moderate to severe synovial thickening and siderotic synovitis (Figure 1). The medial and lateral compartments of the tibiofemoral joint showed extensive, severe cartilage thinning and loss with subchondral cysts and edema. The anterior cruciate ligament (ACL) appeared lax and a partial tear was likely (Figure 2A). The posterior cruciate ligament (PCL) also appeared lax (Figure 2B). The medial and lateral meniscus showed mild extrusion and complex degenerative tears. These findings suggest severe osteoarthritis/hemophilic arthritis of the medial and lateral compartments of the tibiofemoral joint and patellofemoral joint with siderotic synovitis. (While the radiological diagnosis I received was osteoarthritis, the clinical diagnosis was of hemophilic arthropathy.)

Since the time of developing severe arthropathy, I have noticed a slight decrease in bleeding episodes from two to three every year to only three knee bleeds from 2018 to 2021 (two in the left knee and one in the right). During this time, I was able to continue physical therapy on my knees and gain muscle mass and strength around the knees, which drastically reduced the symptoms of arthropathy.

In 2018, I experienced no right elbow arthritis (as indicated by X-rays) but continued to have multiple bleeds in the joint, rendering the muscles weak. In 2020, I started developing a slight flexion deformity in my right elbow with more bleeding episodes than normal, sometimes twice monthly. I was diagnosed with moderate arthritis in my right elbow in 2021 by X-ray. As I continued to experience increased bleeding episodes, I was not able to continue with regular physical therapy of the elbow, presenting with increased loss of muscle mass and strength, and moderate atrophy of muscles around the elbow.

Comparing the knee joints and right elbow shows a clear pattern of degenerative joint disease of all three joints but contrasting patterns of bleeding while having different changes in musculature and vasculature.

## Discussion

The case history described the differing bleeding patterns in the knees and the right elbow due to being in different stages of degenerative joint disease. Many contributing changes due to hemophilic arthropathy and its stages lead to different outcomes. The most common features of hemophilic arthropathy are joint space narrowing and extensive remodeling of subchondral bone [2].

Moderate arthropathy

During early hemarthroses in the knee joint, erythrocytes in the joint space are destroyed by synoviocytes and macrophages. This leads to the expression of oncoproteins in response to hemosiderin, resulting in synovial hypertrophy. Synovial hypertrophy creates a hypoxic environment, causing a rise in hypoxia-inducible factor (HIF), which induces increased expression of the pro-angiogenic mediators vascular endothelial growth factor-A (VEGF-A) and stromal-cell derived factor 1α (SDF-1α), as well as pro-matrix metalloproteinases (pro-MMP) [3].

VEGF-A stimulates synovial neoangiogenesis. Simultaneously, plasmin-mediated conversion to MMPs results in glycosaminoglycan (GAG) release from the cartilage matrix, leading to cartilage and subchondral bone destruction. The combination of synovial hypertrophy and fragile blood vessels makes the joint susceptible to more bleeding, leading to vicious cycles of re-bleeding and more damage [4-6].

Hemophilic arthropathy differs from osteoarthritis and rheumatoid arthritis in that it progresses due to re-bleeding in the joint rather than systemic inflammation (rheumatoid arthritis) or aging processes (osteoarthritis). The changes are closer to osteoarthritis than rheumatoid arthritis due to similar mechanisms and mediators but differ due to triggering factors [3].

Severe arthropathy

During the late (severe) stages, hemophilic arthropathy shows similar changes to osteoarthritis of the joints. Just like osteoarthritis, joint inflammation contributes to the state of hypercoagulation and hypofibrinolysis locally in the joint. This causes venous thrombosis, joint thrombosis, osteocyte necrosis, and subchondral bone remodeling, leading to decreased vasculature and perfusion to the joint. As a result, there are fewer bleeding episodes in the joint [2].

The author of this case report had radiosynovectomies of both knees. A radiosynovectomy is a minimally invasive procedure for the control of synovitis, which involves an intra-articular injection of radioactive colloids that induce necrosis and fibrosis of the hypertrophic synovial membrane [7]. As bleeding episodes decreased, it led to better and continued physical therapy without interruption, leading to better outcomes like increased muscle strength and a better quality of life. This allowed for better outcomes when hemophilic arthropathy developed, compared to the elbow.

The increased bleeding surrounding the elbow can be attributed to having moderate joint disease, increased loss of muscle mass and strength, and having an intact vasculature that has not been destroyed by severe arthropathic changes.

The decreased bleeding in the knee joints can be explained by severe joint disease, loss of vasculature surrounding the joints, better muscle strength and mass, and a surgical history of radiosynovectomies of both knees.

## Conclusions

The most common manifestation of hemophilia is hemarthroses in joints, while the most common chronic complication is hemophilic arthropathy. It is imperative to understand how both of these affect each other. As personal experiences show that the pattern of bleeding episodes can vary wildly depending on the stage of joint destruction, this particular pattern of disease requires more research to reach a definitive correlation. Better research on this topic could help both the patient and the clinician get better outcomes and quality of care.
